# Selection of the Reference Gene for Expression Normalization in *Salsola ferganica* under Abiotic Stress

**DOI:** 10.3390/genes13040571

**Published:** 2022-03-24

**Authors:** Shuran Wang, Sheng Zhang

**Affiliations:** 1 Xinjiang Key Laboratory of Biological Resources and Genetic Engineering, College of Life Science and Technology, Xinjiang University, Urumqi 830046, China; shuranw111@163.com; 2 College of Forestry, Northwest A&F University, Xianyang 712100, China

**Keywords:** *Salsola ferganica*, abiotic stress, reference gene, qRT-PCR

## Abstract

*Salsola ferganica* is a natural desert herbaceous plant in the arid area of western and northwestern China. Because of its salt tolerance and drought resistance, it is of great significance in desert afforestation and sand-fixing capacity. There has been much research on the genes involved in plants under desert stresses in recent years. The application of the best internal reference genes for standardization was a critical procedure in analyzing the gene expression under different types. Even so, the reference gene has not been reported in the application of gene expression normalization of *S. ferganica*. In this study, nine reference genes (*TUA-1726*, *TUA-1760*, *TUB*, *GAPDH*, *ACT*, *50S*, *HSC70*, *APT*, and *U-box*) in *S. ferganica* were adopted and analyzed under six different treatments (ABA, heat, cold, NaCl, methyl viologen (MV), and PEG). The applicability of candidate genes was evaluated by statistical software, including geNorm, NormFinder, BestKeeper, and RefFinder, based on their stability values in all the treatments. These results indicated that the simultaneous selection of two stable reference genes would fully standardize the optimization of the normalization research. To verify the feasibility of the above internal reference genes, the CT values of AP2/ERF transcription factor family genes were standardized using the most (*ACT*) and least (*GAPDH*) stable reference genes in *S. ferganica* seedlings under six abiotic stresses. The research showed that *HSC70* and *U-box* were the most appropriate reference genes in ABA stressed samples, and *ACT* and *U-box* genes were the optimal references for heat-stressed samples. *TUA-1726* and *U-box* showed the smallest value in gene expression levels of cold treatment. The internal reference groups of the best applicability for the other samples were *U-box* and *ACT* under NaCl treatment, *ACT* and *TUA-1726* under MV stress, *HSC70* and *TUB* under PEG treatment, and *ACT* in all samples. *ACT* and *U-box* showed higher stability than the other genes based on the comprehensive stability ranking of RefFinder, as determined by the geometric mean in this study. These results will contribute to later gene expression studies in other closely related species and provide an important foundation for gene expression analysis in *S. ferganica*.

## 1. Introduction

Chenopodiaceae is one of the largest families of angiosperms. Because of its tolerance to drought, salt, and cold, Chenopodiaceae plants can be found in warm and cold zones, semiarid and saline alkaline areas, and the Gobi Desert in Xinjiang, Gansu, and Qinghai provinces, and Chenopodiaceae plants are mostly halophytic or xerophytic [1]. To date, 184 species of Chenopodiaceae [2] belonging to 38 genera have been found in China. *Salsola ferganica* is an annual herb of Chenopodiaceae that has the characteristics of salt tolerance, drought tolerance, and alkaline tolerance. In the process of seed germination and seedling establishment, external environmental factors such as cold, heat, and salt play an important role. Because *S. ferganica* plants have lived in harsh environments for a long time, they have evolved some morphological and physiological adaptations for survival [3]; for example, the leaves are fuzzy, and the seeds are spiral-shaped and have wings. The characteristics of the plant have very important ecological and scientific research value for the restoration of desert vegetation and the study of stress resistance mechanisms [4]. This unique adaptation to the desert environment may play an indispensable part in the expression of some functional genes. Therefore, it is particularly important to screen for stress-tolerance genes at the transcriptome level.

qRT-PCR is an experimental means used to determine the levels of specific products by continuously detecting the change in the fluorescence signal during PCR index amplification, and we can infer the initial amount of the target gene product according to this method. This technology not only realizes both qualitative and quantitative results but also greatly improves the specificity, sensitivity, and accuracy of gene expression detection compared with ordinary PCR [5,6]. This technique is also widely used in gene expression research because it can effectively solve the contamination problem of ordinary PCR. Nevertheless, the data of qRT-PCR are inevitably affected by many reasons, including the integrity of the cDNA template, primer specificity, annealing temperature [7,8,9]. The internal reference gene used in the normalization method can correct the experimental error. Until now, few studies on gene expression in *S. ferganica* have yielded only a few genes as reference genes for normalizing experimental data. Accordingly, it is particularly significant to choose the most stable and active internal reference genes.

Stable expression is the basic principle of screening internal reference genes in any environment [10], such as under different treatments, within different structures, and across different growth phases. Hence, the screening of appropriate reference genes for the study of new species is the beginning of the application of qRT-PCR in the experimental scheme [11]. Because the protein product encoded by a housekeeping gene is necessary to maintain the basic function of cells [12,13], internal reference genes are mainly screened from housekeeping genes due to their stable expression in various samples. At present, several statistical software programs, such as geNorm [14], NormFinder [15], BestKeeper [16], and RefFinder, have also been designed to judge the applicability of the internal housekeeping genes by use of normalization data. These software programs use different calculation methods, varying greatly in the stability rank of candidate genes. Therefore, we referred to the geometric mean method to calculate the comprehensive ranking of the stability of each candidate gene to select the best reference gene.

In this article, we selected nine internal reference genes commonly used for stability evaluation. These genes are α-tubulin (*TUA-1726* and *TUA-1760*), U-box domain-containing protein (*U-box*), actin (*ACT*), glyceraldehyde-3-phosphate dehydrogenase (*GAPDH*), heat shock protein 70 (*HSC 70*), adenine phosphoribosyl transferase-like protein (*APT*), β-tubulin (*TUB*), and 50S ribosomal protein (*50S*). The stable expression of these genes under different stresses (ABA, drought, NaCl, heat, cold, and methyl viologen (MV)) was analyzed, and the stability of the internal reference genes in *S. ferganica* was comprehensively evaluated by geNorm, NormFinder, BestKeeper, and RefFinder software. In addition, to further verify the suitability of these genes as internal reference genes, we selected the most stable internal reference gene (*Actin*) and the least stable internal reference gene (*GAPDH*) in *S. ferganica* and provided detailed expression research of eight AP2/ERF TF family genes as an example for data normalization. Thus, this study provides useful values and beginning points for screening internal reference genes for expression research using the qRT-PCR method in *S. ferganica* and lays a theoretical foundation for further research on desert plants.

## 2. Materials and Methods

### 2.1. Plant Materials and Treatments

In this paper, the seeds of *S. ferganica* were collected near group 103, Wujiaqu, Xinjiang (44°19′ N, 86°57′ E; 429 mH). *S. ferganica* seeds of the same size with full grains and no diseases and pests were cultured in a 9 cm diameter Petri dish covered with two layers of filter paper; 8 mL of distilled water was added to each dish. Then, the *S. ferganica* seeds were cultivated under 16 h/8 h (light /dark) and 25 °C/18 °C for 5 days in artificial climate equipment (RGX400E, Taisite, Tianjin, China). Seed germination was observed every day.

Under the cold- and heat-treated samples, the *S. ferganica* seedlings were cultivated in 4 or 38 °C, respectively. For salt treatment, *S. ferganica* seedlings were treated with 100 mM/L NaCl (>99.5%, 7647-14-5, Jinhuada, Guangzhou, China). For ABA (≥98%, 21293-29-8, Solarbio, Beijing, China) and MV (≥98%, 1910-42-5, Macklin, Shanghai, China) treatment, *S. ferganica* seedlings were grown under 100 μmol/L of either chemical with a 16/8 h day/night light cycle, and groups of seedlings were placed in PEG6000 (10%) (25322-68-3, Kermel, Tianjing, China) solutions in the growth chamber for the drought treatment. All stressed *S. ferganica* seedlings were sampled at 4 time points: 0, 1, 3, and 6 h after treatment. All materials were quickly frozen in liquid nitrogen and then stored at −80 °C for RNA extraction and gene expression analysis. Three biological replicates were set for each treatment, and each replicate included at least six seedlings.

### 2.2. RNA Extraction and cDNA Synthesis

In this chapter, the samples stored in liquid nitrogen were reduced to a fine powder. Total RNA from all samples was extracted by an Omega Total RNA Extraction Kit (Omega Bio-Tek, Beijing, China), and the RNA samples were quantified by an ultramicrospectropho-tometer (Epoch^TM^, Bio-Tek, Santa Clara, CA, USA). The absorbance at 260/280 nm was 1.8–2.2, and the integrity of RNA was assessed by 1% agarose gel electrophoresis. The extracted RNA was inverted into first-strand cDNA according to EasyScript^®^ First-Strand cDNA Synthesis Super-Mix (TransGen Biotech, Beijing, China), and the reverse cDNA was diluted 10× for subsequent experiments and refrigerated at −20 °C for standby.

### 2.3. PCR Primer Design

In a previous study, transcriptome data were analyzed, and internal reference gene sequences were obtained. All the internal reference gene primers were designed with Primer 5.0 software [17]. The primers were synthesized by Shanghai Sangon Biotech Company and stored at 4 °C.

### 2.4. Detection of Amplification Efficiency and Selection of Reference Genes

According to previous studies, a total of 9 housekeeping genes (*TUA-1726*, *TUA-1760*, *TUB*, *GAPDH*, *ACT*, *50S*, *HSC 70*, *APT*, *U-box*) were selected for analysis of expression stability under abiotic stress in *S. ferganica*. The amplification products were diluted with stock solution 10, 10^2^, and 10^3^ times for qRT-PCR amplification. All data were plotted along a standard curve, the amplification efficiency of primers was analyzed. The original CT value was used to calculate primer amplification efficiency (E) and correlation coefficient (R^2^) respectively with the following formula: E = 10^−1/slope^ − 1 [18], where the slope was derived from the regression equation and was calculated using Excel linear regression reference genes [19]. The primer data of the candidate housekeeping genes are given in Table 1.

### 2.5. qRT-PCR Analysis

qRT-PCR was performed according to the requirements of the 2 × SYBR Green qPCR mix reagent Manual of Beijing Aidlab Biotechnologies Company (Aidlab, Beijing, China). The fluorescence quantitative reaction of 9 internal reference genes was completed on a LightCycler^®^ 96 fluorescence quantitative instrument (LightCycler^®^ 96 Instrument, Roche, IN, USA) with a 20 μL reaction system as follows: 2 × SYBR qPCR Mix 12.5 μL; ddH_2_O 10.5 μL; reverse primers and forward primers, respectively, 0.5 μL; and cDNA template, 1 μL. Each sample was tested by the three-step method in the PCR cycle. The experimental system was as follows: predenaturation at 94 °C for 30 s; denaturation at 94 °C for 20 s; annealing at 60 °C; and extension for 30 s at 72 °C over 40 cycles. After the completion of the reaction system and program, the data reading was automatically completed by fluorescence quantitative PCR.

### 2.6. Gene Expression Stability Analysis

All CT values gained from qRT-PCR were used to evaluate the stable values of all selected genes using the above three software (geNorm, NormFinder, and BestKeeper), and combined with the analysis results, the comprehensive ranking of candidate genes in different treatments was calculated by RefFinder according to the formula Q = 2^−ΔCT^ (ΔCT = CT_max_ − CT_min_ in samples). GeNorm calculates the expression stability by analyzing the Q value after input processing. An M value < 1.5 is considered within the range of stability, and the stability becomes increasingly stable with a decrease in the M value; conversely, the gene stability decreases with an increase in the M value. The principle of NormFinder analysis is similar to the principle of geNorm. Q value is used for calculation. Similarly, as the value decreases, the relative expression level of genes becomes more stable. In NormFinder, the linking of the samples set between intragroup and intergroup variations was applied to the calculation of gene expression stability. The CT value of the original data was directly used in the best keeper analysis to obtain the mean and standard deviation. Finally, the above data were compared and analyzed through the RefFinder website to obtain the most stable internal reference gene.

### 2.7. Stability Evaluation of Candidate Genes

To evaluate the applicability of the selected optimum gene, eight AP2/ERF TF family genes that play an indispensable role in plant hormone signals and the regulation of gene expression related to biological and abiotic stress [20,21] served as indicator genes. For the salt stress treatments, the seedlings were grown in pots under 0, 100, 200, 300, and 400 mmol/L NaCl for 0, 4, or 24 h. These procedures, total RNA extraction, cDNA synthesis, transcription factor family gene primer design and verification, and qRT-PCR analysis of expression, were performed using processed samples. Three technical and biological replicates were carried out in this study.

## 3. Results

### 3.1. Primer Specificity and Amplification Efficiency of Candidate Reference Genes

**Figure 1:**
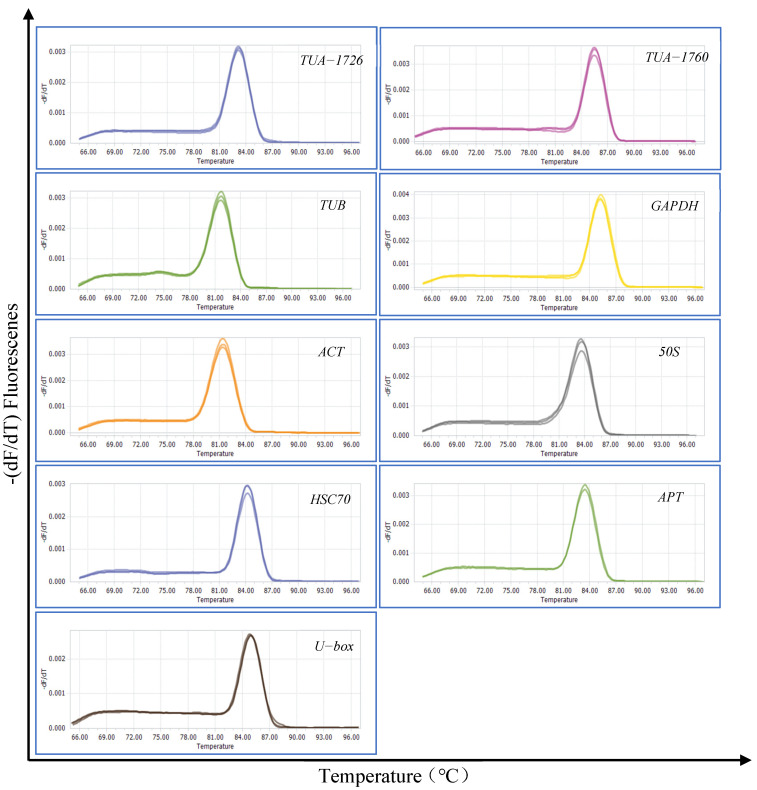
Melting curves of 9 candidate reference genes.

Total RNA was quantified by gel electrophoresis and the OD260/280 value. The successful extraction was that two complete and clear RNA bands (28S and 18S) was displayed, and the OD260/280 value was between 2.0 and 2.2. Nine genes (*TUA-1726*, *TUA-1760*, *TUB*, *GAPDH*, *ACT*, *50S*, *HSC 70*, *APT*, and *U-box*) were selected and used as candidate reference genes. Data about these nine candidate genes and primers are presented in Table 1. The specificity of the primers was verified by agarose gel (1%) electrophoresis and dissolution curves (Figure S1). The gel electrophoresis showed that all primers of the nine reference genes showed a single and bright band, and the melting curve analysis also showed a single peak (Figure 1). The dissolution curves showed an acceptable amplification efficiency range of 90–110%, and the correlation coefficient (R^2^) ranged from 0.99 to 1 (Table 1). The above results meet the requirements of candidate reference gene screening.

### 3.2. Relative Expression of Candidate Genes in Different Treatments

**Figure 2:**
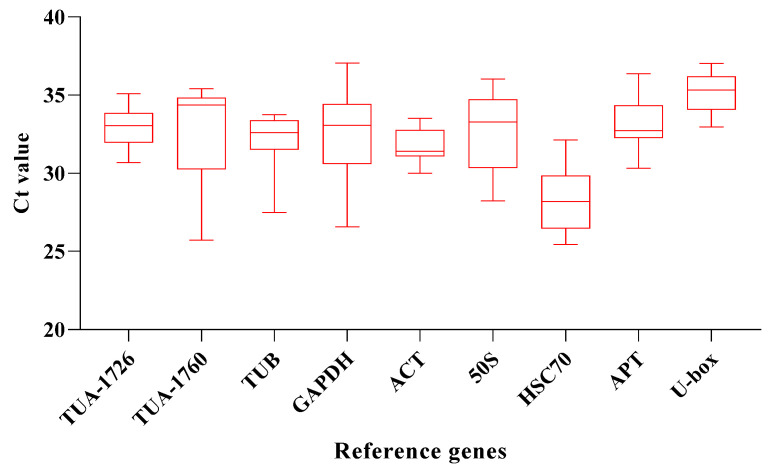
Average CT value of 9 candidate genes. The line across the box represents the median. The boxpoints out the 25th and 75th percentiles. Whiskers represent the maximum and minimum values across all samples.

qRT-PCR assays were used to measure the transcriptional abundance of the 9 housekeeping genes across 24 samples, including six abiotic stress conditions (ABA, drought, NaCl, heat, cold, and MV) and 4 different time treatments (0, 1, 3, and 6 h). The result pointed out that the average Ct values of these housekeeping genes were between 26 and 36, with most of them ranging from 30 to 33 in all plant samples. The expression of *HSC70* was the highest in the control, and the Ct value was 26.46. Accordingly, the gene abundance level was also the highest. For candidate genes, the average Ct value was between 28 and 35, and the Ct value of *U-box* is the highest, reaching 35.04, which means that it has the lowest expression level than other internal reference genes. The average Ct of *HSC70* was the lowest, only 28.04, which means that the expression of *HSC70* is the highest compared with other internal reference genes (Figure 2). The uneven results also indicated that there was no significant expression regularity of different reference genes under different stresses, which needs to be further evaluated by stability analysis software.

### 3.3. Stability Evaluation of Candidate Genes

In this paper, the gene expression of *S. ferganica* was quantified by qRT-PCR under six stresses (i.e., ABA, drought, NaCl, heat, MV, and cold). Then, four softwares (geNorm, NormFinder, BestKeeper, and RefFinder) were calculated to analyze the size of CT value, and the most suitable internal reference genes were further selected.

#### 3.3.1. geNorm Analysis

**Figure 3:**
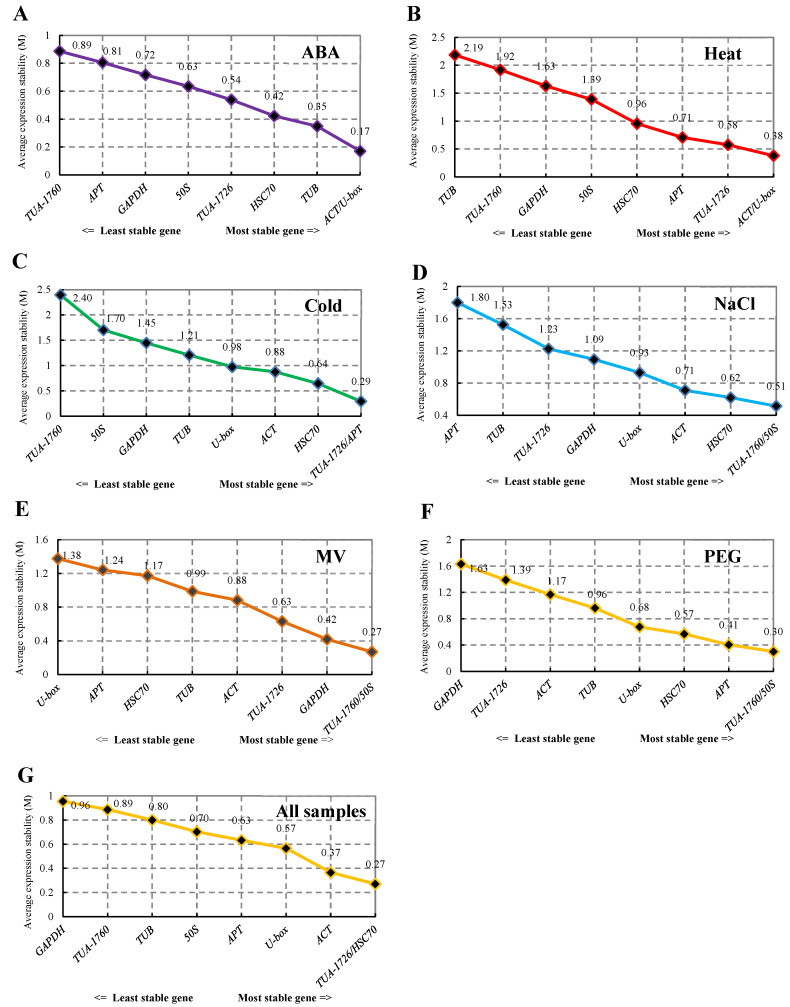
Expression stability values of 9 internal reference genes analyzed by geNorm software. (**A**) Internal reference genes in the ABA treatment group. (**B**) Internal reference genes of heat-treated samples. (**C**) Stability ranking of internal reference genes in all samples treated with cold. (**D**) Internal reference genes from NaCl-treated samples. (**E**) Reference genes derived from MV. (**F**) PEG-treated samples representing drought treatment. (**G**) All samples used in this study.

The expression stability of the nine housekeeping genes was analyzed using geNorm software. The value of M was the standard for ranking the expression stability of screened genes. With the increase in M value, the gene showed increasingly poor stability, and the average pairwise variation is for each candidate gene compared with all other genes [14]. The stability sequencing of internal reference genes of *S. ferganica* was calculated by geNorm under six abiotic stresses (Figure 3). As illustrated in Figure 3, the M values from six treatments and all samples were arranged from large to small. *ACT* and *U-box* (M = 0.17) had lower M values than other genes. thus, they were the most stable under ABA treatment. The most stable genes were *ACT* and *U-box* under heat treatment, which was the same as ABA treatment. While *TUB* was the most unstable, with an M value of 2.19. As the cold stress group, the most stable internal reference genes were *TUA-1726* and *APT*, with an M value of 0.08. The stability of *TUA-1760* and *50S* was the highest in the NaCl, MV, and PEG treatments, but *APT*, *U-box,* and *GAPDH* were analyzed as the internal reference genes with the worst stability for these three treatments. When all 24 samples were analyzed together, the combination of *TUA-1726* and *HSC70* was the smallest M value, while *GAPDH* was the most unstable (Figure 3).

**Figure 4:**
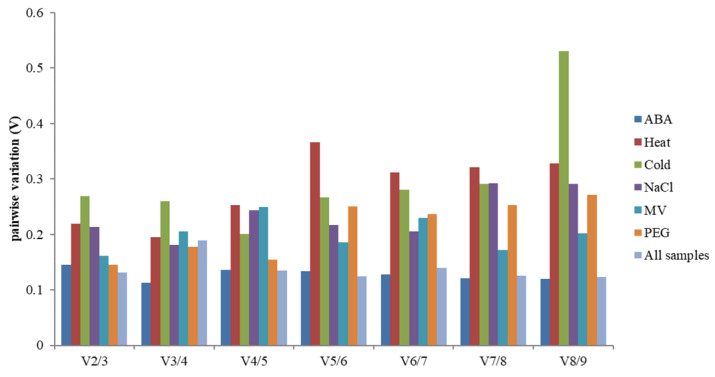
The V value analysis of 9 candidate genes under abiotic stress by geNorm. Pairwise variation (Vn/Vn + 1) analysis of 9 candidate reference genes analyzed in six sample subsets.

In addition, screening the number of the most suitable reference genes needed for experimental standardization, the best-paired variation value (Vn/Vn + 1) [14] was analyzed using geNorm. This calculation method is calculated for selecting the number of genes by standardized gene expression. If the value exceeds 0.15, n + 1 is the most appropriate number of internal reference genes. Such as, the left and right values of the ABA treatment group were lower than 0.15, which showed that the two candidate housekeeping genes standardized gene expression (Figure 4).

#### 3.3.2. NormFinder Analysis

NormFinder software is a basic application plug-in unit installed in Microsoft Excel, which can screen the internal reference gene with the best stability among all candidate genes; this software package analyses the stability value of expression based on the estimation of intragroup variation and intergroup variation [22] and sorts the candidate genes. Similar to geNorm software, lower stability values indicate a more appropriate reference gene [23]. The stability values of each internal reference gene in each stress group are shown in Table 2.

In ABA-treated samples, the two most suitable genes (*HSC70* and *TUA-1726*) and the least stable genes (*APT* and *TUA-1760*) were identified in NormFinder. Among the heat-treated samples, *ACT* had the lowest stable value, with a value of 0.19, which indicated that it expresses the greatest stability at the expression level. For the cold stress samples, *TUA-1726* is the most suitable internal reference gene, and *TUA-1760* was ranked as the most active gene. U-box was calculated as the optimal internal reference gene under NaCl stress, while the stable value of *APT* is the largest of all genes. About the MV treatment, *ACT* was still in the top position; on the contrary, *U-box* was the most active. Moreover, *TUB* and *HSC70* were recognized as the most suitable candidate genes, while *GAPDH* was the least stably expressed gene in PEG treatments. For all stress samples, *ACT*, *TUA-1726,* and *HSC70* were calculated to be the three stable expression genes. On the contrary, *GAPDH* was the most unstable, which was the same as the conclusion of geNorm above. This part concludes that *ACT* was the optimum reference gene compared with all other genes. This result further verifies the stability of *ACT*, and *GAPDH* was likely the least stable housekeeping gene.

#### 3.3.3. BestKeeper Analysis

BestKeeper analysis the original CT values of all samples and ranks the stability of candidate housekeeping genes by determining the SD and CV values of each candidate gene [24]. The stability of gene expression changed inversely with the SD value. The results of the stability analysis of different treated samples from highest to lowest are shown in Table 3. Accordingly, the U-box was the best reference gene under ABA, heat, and cold stresses, and the lowest CV ± SD values of the three treatments were 1.434 ± 0.507, 1.842 ± 0.646, and 1.484 ± 0.53, respectively. The sequence of gene expression stability in the NaCl treatment was *TUA-1726 > U-box > TUB > ACT > APT > TUA-1760 > HSC70 > GAPDH > 50S*. *TUA-1726* TUA was the housekeeping gene with the lowest SD value, and *50S* was the most unstable gene. *HSC70* was the best housekeeping gene under MV stress and PEG stress. The order of gene stability across all samples was *U-box > APT > TUB > ACT > HSC70 > TUA-1726 > 50S >TUA-1760 > GAPDH*. In conclusion, the optimum reference gene calculated for all the above samples was likely *U-box*. Because the above results are heterogeneous under different treatments, this cannot be taken as the final result. Therefore, additional analytical tools should be used for normalization.

#### 3.3.4. RefFinder Analysis

**Figure 5:**
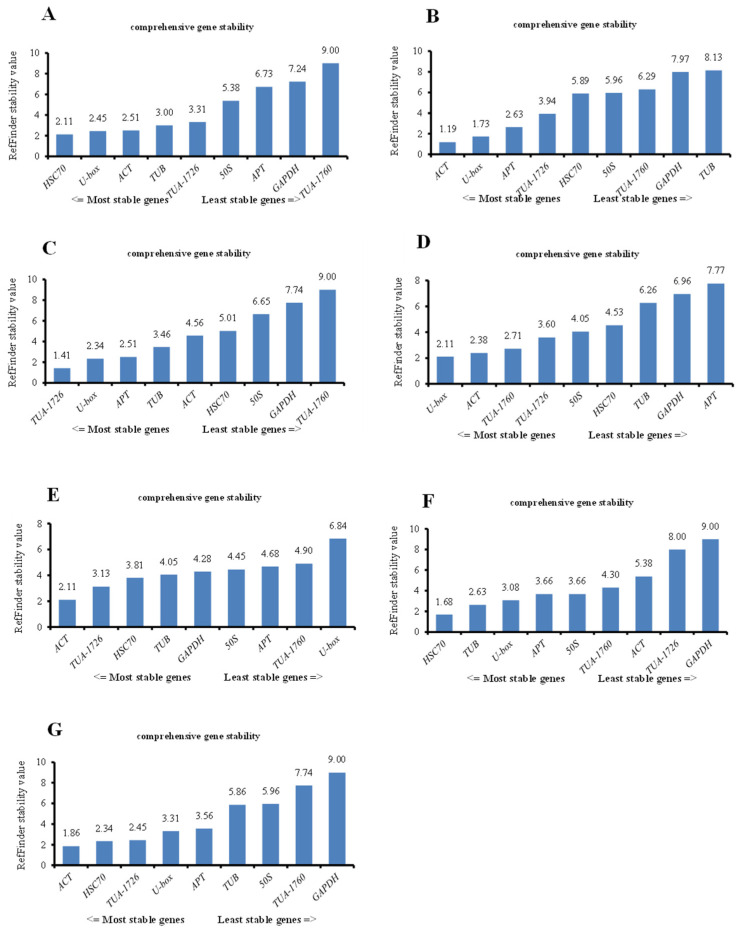
Stability ranking of all reference genes in different treatments of *S. ferganica* calculated by the RefFinder in all samples. (**A**) Internal reference genes in the ABA treatment group. (**B**) Internal reference genes in heat-treated samples. (**C**) Stability ranking of internal reference genes in all samples treated with cold. (**D**) Internal reference genes from NaCl-treated samples. (**E**) Reference genes derived from MV. (**F**) PEG-treated samples representing drought treatment. (**G**) All samples used in this study.

In the above analyses, we analyzed the optimum internal reference genes for different treatments based on the above three software commonly, but the analysis ranking of the three softwares was different. To standardize the analysis and to verify whether the software produces heterogeneous results, we used the RefFinder website to comprehensively compare and analyze the data from the above three software programs and rank the stability of all genes through comprehensive analysis. The gene with the highest gene expression stability was *ACT* for heat, MV, and all samples. Insides, the most stable internal reference gene in the other samples was *HSC70* (2.11) under ABA treatment, *TUA-1726* (1.41) under cold stress, *U-box* (2.11) under NaCl treatment, and *HSC70* (1.68) in PEG stress samples (Figure 5). The above results also point out that a single reference gene in the experiment could not standardize the gene expression of plants in all environments and could only be used for one or more abiotic stresses.

Second, we integrated the sequence of the housekeeping genes into RefFinder with different treatments, and the most suitable internal reference gene was selected based on the geometric mean (Table 4). In all treatments, the lowest comprehensive ranking was the best internal reference gene, while the highest ranking was the most unstable. Therefore, *ACT* (2.857) was the best housekeeping gene, followed by *U-box* (3.286) and *HSC70* (3.571). *GAPDH* was the least stable of candidate gene expression, and the geometric mean of its comprehensive ranking was 7.857 (Table 4).

### 3.4. Validation of the Best- and Worst-Ranked Reference Genes

**Figure 6:**
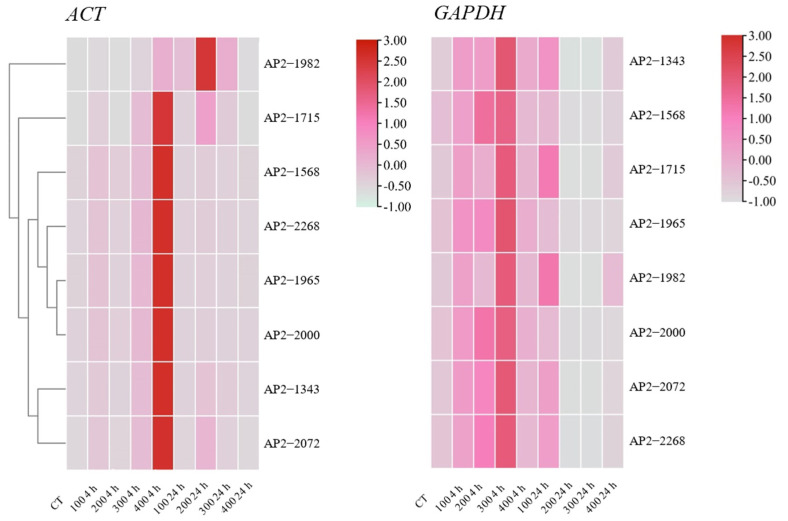
Expression heatmaps of eight transcription factors in *S. ferganica* under NaCl stress. The best (*ACT*) and worst (*GAPDH*) reference genes were used to normalize the expression data. cDNA and the above gene expression analysis were the same samples in this experiment. Samples were collected from five-day-old seedlings subjected to 0, 100, 200, 300, and 400 mmol/L NaCl stress after 0, 4, and 24 h of treatment.

To verify the selection of the optimal housekeeping genes, the expression patterns of eight AP2/ERF transcription factor family genes were changed with 0, 100, 200, 300, and 400 mmol/L NaCl stress treatments for 0, 4, and 24 h. In this article, the most stable and active housekeeping genes from all stress sample sets, *ACT* and *GAPDH*, were used for normalization. Except for AP2-1982, the expression patterns of the other AP2 genes were similar, and all genes showed the highest expression when treated with 400 mmol/L NaCl for 4 h. When normalized to *GAPDH*, the expression pattern was obviously underestimated, and the highest expression was found with 300 mmol/L NaCl treatment for 4 h. Therefore, the gene expression patterns in AP2/ERF transcription factor family genes differed greatly from those of the reference genes (Figure 6). Hence, screening an appropriate reference gene was the first step in the comprehensive research of stress-tolerance genes.

## 4. Discussion

In nature, abiotic stress environment continuously affects the growth and development of plants, such as drought, high temperature, cold and excessive salt in the soil [25]. Desert plants play an irreplaceable ecological role in desert ecosystems because of their salt-alkali tolerance and drought tolerance [26]. *S. ferganica* is a crucial desert plant, and with the increase in demand, there is a need to comprehensively analyze its expression mechanisms under stress from morphological, physiological, and molecular biological points of view, which will help promote the reproduction of desert plants and the governance of desert areas.

With the increasing interest in research on stress-related genes in desert plants, the demand for new high-throughput technologies such as genomics, transcriptomics, and proteomics has also increased [27,28]. SMRT-seq and qRT-PCR are important tools for determining the expression of stress-related genes and explaining their effectiveness in all stages of growth and development. qRT-PCR is a highly reliable method to verify targeted differential gene expression. Due to its high efficiency and sensitivity at the molecular level, gene expression research is generally carried out by qRT-PCR [29]. However, a stable housekeeping gene is a prerequisite for accurate standardization of expression data by qRT-PCR [30,31]. In most experiments, a single housekeeping gene is currently selected to evaluate qRT-PCR data [32]. However, it is found that using two or more internal references for normalization will produce a more accurate and stable relative expression level. Although several internal reference genes have been used to analyze the expression of related genes in *Salsola laricifolia* under drought stress, *β-actin* is the most suitable internal reference gene [33]. However, the storage of internal reference genes under abiotic stress was still insufficient. Therefore, high-throughput sequencing-based transcriptome data from SMRT-seq are essential for meticulously selecting candidate internal reference genes for expression normalization, but this gene expression analysis needs further expression stability verification under relevant environmental stress conditions [10].

The common methods for preliminary screening of housekeeping genes are mainly based on the functions of housekeeping genes, such as participating in protein coding, cell signaling, morphogenesis, and so on [34]. For instance, *ACT* and *TUB* genes are mainly involved in the main components of the cytoskeleton synthesis; Likewise, *GAPDH*, *EF-1α,* and *UBQ* genes can play significant roles in the material metabolism and life activities of organisms [35]. The internal reference genes should not be limited to ideal states, and their expression levels should be the same across conditions [36]. Subsequent research pointed out that the expression level of the internal reference gene can be affected by the species, environment, physiology, and developmental stage [37]. Thus, no internal reference gene was continuously and stably expressed under all experimental conditions. For instance, genes such as *ACT*, *GAPDH,* and *U-box* are thought to be expressed differently in different species and under different environmental stress [38,39,40]. *PP2A* and *GAPDH* are the most suitable internal reference genes in sorghum across different structures and under abiotic stresses [11]. However, the stable expression of *GAPDH* in all treatment samples was poor in this study (Figure 5, Table 4). Similar results were also verified in *Anemone flaccida* [41]. In this research, the geNorm results indicated that *ACT* and *U-box* were the most stable genes under ABA and heat treatment at the seedling stage, while *50S* and *TUA-1760* were the optimal internal reference genes under NaCl, MV, and PEG treatment (Figure 3). Therefore, the qRT-PCR data of a species under specific conditions must be standardized using internal reference genes before conducting gene expression research [42].

In this study, samples of *S. ferganica* from six abiotic stress treatments were collected for screening nine candidate internal reference genes. Several different calculation methods were used to comprehensively analyze the normalization of gene expression data to determine the order of expression stability for internal reference genes under each treatment. The results showed that there were some differences in the expression stability ranking of candidate internal reference genes based on the different calculation methods. After consulting the literature, the analysis results were found to be different under different calculation methods [43,44,45]. Since each method analyzed in the study also has advantages and disadvantages, using only one of these methods is not enough to obtain unbiased results. Therefore, more than three methods are recommended for analysis and calculation. We used the ranking methods of geNorm, NormFinder, BestKeeper, and RefFinder to calculate the geometric average for the final stability consensus ranking in the paper. Based on the comprehensive RefFinder ranking results calculated using the three software tools and the ΔCt method, the combinations of *HSC70* and *U-box*, *ACT* and *U-box*, *TUA-1726* and *U-box*, *U-box* and *ACT*, *ACT* and *TUA-1726*, and *HSC70* and *TUB* were recommended as reference genes for analyses of ABA, heat, cold, NaCl, MV, and PEG, respectively (Figure 5). *ACT* and *U-box* were recommended as reference genes for the analyses of all samples together (Table 4). These rankings also confirmed that there were differences in the expression levels of some housekeeping genes in plants based on space-, time-, and environment-dependent patterns. This internal reference gene screening process under multiple culture conditions can be used as a guide for researchers to study desert plants.

AP2/ERF (APETALA 2/ethylene-responsive element binding factor) is a large superfamily of transcription factors in various plants. The expression of AP2/ERF transcription factors can affect the expression of functional genes related to abiotic stress tolerance, such as drought [46], high salt [47], and cold [48]. Two reference genes (*ACT* and *GAPDH*) identified in the existing research were used to analyze the expression profile of eight transcription factor genes in *S. ferganica* (AP2-1982, AP2-1715, AP2-1586, AP2-2268, AP2-1965, AP2-2000, AP2-1343, and AP2-2072). The eight AP2/ERF transcription factor family genes showed different specific expression patterns under standardization with the most stable and active internal candidate genes and showed an increase in expression after salt treatment (Figure 6). This finding indicates that AP2/ERF genes play a vital part in the salt tolerance mechanism of *S. ferganica* and shows that it is necessary to screen housekeeping genes at the molecular level. The above discussion shows that our research approach is feasible; that is, under a variety of stresses, we used a variety of calculation methods to screen appropriate internal reference genes for a specific species to normalize qRT-PCR data. The appropriate internal reference genes we found in *S. ferganica* are expected to assist in future gene expression research.

## 5. Conclusions

We calculated the expression stability of nine candidate genes under six abiotic stresses in this research. Inside, *ACT* was the best stable internal reference gene for heat and MV treatment; the housekeeping gene that can best be corrected for target gene about ABA stress and PEG stress was *HSC70*; *U-box* was the optimal gene among the NaCl treatment samples; the most stable internal reference genes in all samples were *ACT* and *HSC70*; the geometric mean was calculated to analyze the comprehensive stability of all internal reference genes. Finally, the most stable internal parameter genes were *ACT* and *U-box*. The above was further verified that different treatments should use different endogenous reference genes to standardize the expression level of the gene of interest. In general, the selection of internal reference genes in this study provides a reference for the related plant biochemical and molecular research of *S. ferganica* under different stress conditions, which will greatly contribute to our exploration of the molecular mechanism of adverse environment tolerance and have important scientific significance for promoting research on desert plants.

## Figures and Tables

**Table 1:** Analysis of *S. ferganica* internal reference genes, primer information, and PCR amplification data.

Gene	Gene Description	Primer Sequences (5’→3’)	Amplification Length/bp	Amplification Efficiency/%	R^2^
*TUA-1726*	α-tubulin	GTGGCACTGGTTCTGGACTTG	108	98.35	0.9977
		TTGAAACTTGAGGAGACGGGTAA			
*TUA-1760*		TCCGCAAGCTCGCTGATA	161	105.82	0.9992
		GGGAGATGGGTAGATGGTGAA			
*TUB*	β-tubulin	TTACACTGAGGGTGCCGAAC	92	90.94	0.9995
		AAACCTGGAATCCTTGAAGACA			
*GAPDH*	Glyceraldehyde-3-phosphate dehydrogenase	CCATCCTCGGCACATTCAAC	146	102.21	0.9934
		TCCTTCAATCACCAAGTCTACGC			
*ACT*	Actin	TTCATCGGAGACGAAGCAGTAG	107	97.89	0.9996
		AACCTTTCCATAGCATCCCAGT			
*50S*	50S ribosomal protein	TTGCTAAGCCTGGTTGCATC	138	95.11	0.9998
		TGTCAGGACCAAACTTCTCAAAT			
*HSC 70*	Heat shock protein 70	CCAATGACAAGGGTAGGCTCT	141	101.78	0.9991
		TCCTCATGTTGTAGGCGTAGTTC			
*APT*	Adenine phosphoribosyl transferase-like protein	AAGGCTGAAGTGGCTGAATGT	127	105.85	0.9908
		TCCTTAAACGGCAGTCTTCTAACT			
*U-box*	U-box domain-containing protein	AACACTTGATTCACGCACCCA	143	95.76	0.9921
		TTGCTTCCATGCTGCCTTTC			

**Table 2:** Expression stability ranking of all candidate genes under 6 abiotic stresses on NormFinder.

Rank	ABA		Heat		Cold		NaCl		MV		PEG		All Samples	
	Gene	Stability	Gene	Stability	Gene	Stability	Gene	Stability	Gene	Stability	Gene	Stability	Gene	Stability
1	*HSC70*	0.126	*ACT*	0.190	*ACT*	0.248	*TUB*	0.374	*ACT*	0.264	*TUA-1726*	0.390	*U-box*	0.246
2	*TUA-1726*	0.173	*APT*	0.325	*TUA-1726*	0.326	*HSC70*	0.590	*TUA-1726*	0.403	*U-box*	0.629	*ACT*	0.295
3	*TUB*	0.512	*U-box*	0.385	*TUB*	0.349	*U-box*	0.717	*HSC70*	0.437	*TUB*	0.671	*TUA-1760*	0.767
4	*50S*	0.595	*TUA-1726*	1.029	*GAPDH*	1.050	*ACT*	0.893	*APT*	0.586	*APT*	0.743	*TUA-1726*	1.158
5	*ACT*	0.617	*50S*	1.930	*APT*	1.058	*APT*	1.200	*50S*	0.596	*50S*	1.534	*HSC70*	1.197
6	*U-box*	0.733	*HSC70*	1.939	*HSC70*	1.068	*50S*	1.279	*U-box*	0.642	*ACT*	1.576	*50S*	1.255
7	*GAPDH*	0.776	*TUA-1760*	2.092	*50S*	1.386	*TUA-1760*	1.610	*TUA-1760*	0.984	*HSC70*	1.604	*GAPDH*	1.619
8	*APT*	0.956	*GAPDH*	2.301	*TUA-1760*	1.529	*TUA-1726*	1.629	*TUB*	1.018	*GAPDH*	2.249	*TUB*	1.955
9	*TUA-1760*	1.061	*TUB*	2.903	*U-box*	1.785	*GAPDH*	2.415	*GAPDH*	1.089	*TUA-1760*	4.746	*APT*	2.586

**Table 3:** Stability ranking of all sample genes calculated by BestKeeper software.

Rank	ABA			Heat			Cold			NaCl			MV			PEG			All Samples		
	Gene	SD	CV	Gene	SD	CV	Gene	SD	CV	Gene	SD	CV	Gene	SD	CV	Gene	SD	CV	Gene	SD	CV
1	*U-box*	0.507	1.434	*U-box*	0.646	1.842	*U-box*	0.530	1.484	*TUA-1726*	0.720	2.263	*HSC70*	0.212	0.807	*HSC70*	0.520	1.924	*U-box*	0.206	0.588
2	*ACT*	0.513	1.639	*ACT*	0.766	2.463	*TUB*	0.719	2.195	*U-box*	0.755	2.089	*APT*	0.227	0.700	*U-box*	0.527	1.534	*APT*	0.411	1.242
3	*TUB*	0.574	1.740	*APT*	0.858	2.631	*ACT*	1.068	3.401	*TUB*	0.793	2.505	*U-box*	0.507	1.509	*APT*	0.705	2.154	*TUB*	0.428	1.333
4	*APT*	0.738	2.214	*TUA-1760*	1.113	3.785	*TUA-1726*	1.302	3.928	*ACT*	1.300	4.056	*ACT*	0.540	1.726	*TUB*	0.711	2.195	*ACT*	0.574	1.827
5	*HSC70*	0.785	2.809	*TUA-1726*	1.198	3.717	*APT*	1.331	3.846	*APT*	1.583	4.826	*TUB*	0.562	1.713	*50S*	0.958	3.127	*HSC70*	0.821	2.926
6	*TUA-1726*	1.047	3.187	*TUB*	1.473	4.914	*HSC70*	1.608	5.487	*TUA-1760*	1.705	5.061	*TUA-1726*	0.964	2.950	*ACT*	1.011	3.244	*TUA-1726*	0.862	2.656
7	*50S*	1.185	3.624	*50S*	1.760	5.731	*50S*	1.778	5.516	*HSC70*	1.709	5.719	*GAPDH*	1.368	4.250	*TUA-1760*	1.087	3.545	*50S*	1.002	3.099
8	*GAPDH*	1.389	4.311	*HSC70*	1.862	6.693	*GAPDH*	2.303	6.765	*GAPDH*	1.796	5.440	*50S*	1.608	4.793	*TUA-1726*	1.483	4.615	*TUA-1760*	1.090	3.402
9	*TUA-1760*	1.515	4.550	*GAPDH*	1.887	6.377	*TUA-1760*	3.473	11.036	*50S*	1.941	5.672	*TUA-1760*	1.708	5.070	*GAPDH*	1.987	6.425	*GAPDH*	1.276	3.989

**Table 4:** Expression stability ranking of 9 candidate genes in all stressed samples analyzed using the geometric mean method in RefFinder.

Gene	*ACT*	*U-Box*	*HSC70*	*TUA-1726*	*TUB*	*APT*	*50S*	*TUA-1760*	*GAPDH*
ABA	3	2	1	5	4	7	6	9	8
Heat	1	2	5	4	9	3	6	7	8
Cold	5	2	6	1	4	3	7	9	8
NaCl	2	1	7	4	6	9	5	3	8
MV	1	9	3	2	4	7	6	8	5
PEG	7	3	1	8	2	4	5	6	9
All samples	1	4	2	3	6	5	7	8	9
Mean	2.857	3.286	3.571	3.857	5.000	5.429	6.000	7.143	7.857

## Data Availability

Not applicable.

## Supplementary Materials

The following supporting information can be downloaded at: https://www.mdpi.com/article/10.3390/genes13040571/s1, Figure S1: Primer specificity and amplicon size. Agarose gel electrophoresis (2.0%) shows amplification of a single PCR product of the expected size for 9 genes (Line 1–9: *TUA-1726*, *TUA-1760*, *TUB*, *GAPDH*, *ACT*, *50S*, *HSC 70*, *APT*, and *U-box*).
