# Supplementary material for: Selection of the Reference Gene for Expression Normalization in Salsola ferganica under Abiotic Stress
Source: Genes (Basel). 2022 Mar 24;13(4):571. doi: 10.3390/genes13040571 (PMC9029158; doi:10.3390/genes13040571)
Supplement: Supplementary file 1 [file genes-13-00571-s001.zip › genes-1630483-supplementary.pdf]

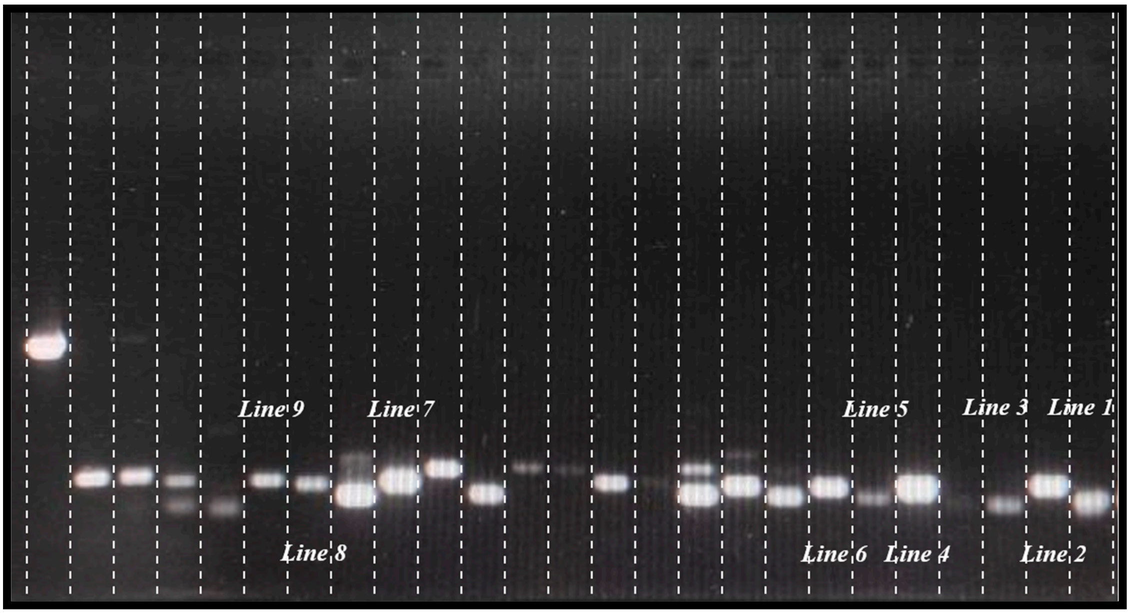

Figure S1. Primer specificity and amplicon size. Agarose gel electro-phoresis (2.0%) shows amplification of a single PCR product of the expected size for 9 genes (Line 1–9: *TUA-1726*, *TUA-1760*, *TUB*, *GAPDH*, *ACT*, *50S*, *HSC 70*, *APT*, and *U-box*).
